# Molecular Mechanisms of Induction of Tolerant and Tolerogenic Intestinal Dendritic Cells in Mice

**DOI:** 10.1155/2016/1958650

**Published:** 2016-02-11

**Authors:** Alex Steimle, Julia-Stefanie Frick

**Affiliations:** University of Tübingen, Institute of Medical Microbiology and Hygiene, Elfriede-Aulhorn-Strasse 6, 72076 Tübingen, Germany

## Abstract

How does the host manage to tolerate its own intestinal microbiota? A simple question leading to complicated answers. In order to maintain balanced immune responses in the intestine, the host immune system must tolerate commensal bacteria in the gut while it has to simultaneously keep the ability to fight pathogens and to clear infections. If this tender equilibrium is disturbed, severe chronic inflammatory reactions can result. Tolerogenic intestinal dendritic cells fulfil a crucial role in balancing immune responses and therefore creating homeostatic conditions and preventing from uncontrolled inflammation. Although several dendritic cell subsets have already been characterized to play a pivotal role in this process, less is known about definite molecular mechanisms of how intestinal dendritic cells are converted into tolerogenic ones. Here we review how gut commensal bacteria interact with intestinal dendritic cells and why this bacteria-host cell interaction is crucial for induction of dendritic cell tolerance in the intestine. Hereby, different commensal bacteria can have distinct effects on the phenotype of intestinal dendritic cells and these effects are mainly mediated by impacting toll-like receptor signalling in dendritic cells.

## 1. Introduction

The mammalian intestinal immune system has to rise to different challenges. On the one hand, it has to tolerate the intestinal microbiota consisting of commensal bacteria, fungi, and other microbes, thereby profiting from beneficial bacterial metabolites and other advantages. On the other hand, pathogen induced infections of the intestine have to be cleared without spacious damage of the intestinal tissue. Since a loss of tolerance to the own microbiota causes chronic inflammation of the gut, efficient sensing of the intestinal homeostasis is crucial to avoid pathophysiological immune responses. In this context, intestinal tolerogenic dendritic cells play a crucial role as key mediators for the maintenance of the intestinal homeostasis. While the main question “how does the host manage to tolerate its own intestinal microbiota?” is pretty simple, the answer is not trivial.

Here, we want to focus on (1) the molecular mechanisms that might contribute to the induction of tolerogenic DCs in the intestine and (2) the potential clinical applications arising from these findings for the treatment of chronic inflammatory disorders of the gut: inflammatory bowel diseases.

## 2. Intestinal Dendritic Cells: Subsets and Biological Functions

Dendritic cells (DCs) comprise a heterogeneous leukocyte population of different developmental origin and with distinct surface markers and biological functions. DCs originate from blood monocytes or a common DC progenitor in the bone marrow under steady-state conditions. The differentiation into DCs relies on local presence of GM-CSF [1]. DCs in general are utterly specialized antigen presenting cells (APCs) which are able to induce a variety of different immune responses. They are the most important cell type connecting the innate immune system with adaptive immune responses [2]. DCs patrol almost all lymphoid and nonlymphoid organs and meld properties of the innate and adaptive immunity and therefore link these two mechanistically distinct branches of the immune system [3]. Furthermore, DCs play a pivotal role in mediating a protective adaptive immunity against pathogens while maintaining immune tolerance to self-antigens. Their crucial role for mediating self-tolerance is confirmed by the observation that DC depletion leads to a loss of self-tolerance and results in myeloid inflammation and the induction of autoimmune processes [4].

The gut-associated lymphoid tissue (GALT) is the largest immune organ of the body. The GALT has to ensure that there is a dynamic balance between protective immunity by fighting pathogens and regulatory mechanisms to prevent autoimmunity [5]. Since the GALT is constantly exposed to large amounts of luminal antigens like food metabolites, foreign pathogens, and commensal microbes, this balance has to be well adjusted in order to create homeostatic conditions in the intestine. Dendritic cells are hereby the key players for maintaining intestinal homeostasis [6]. They are spread out in the connective tissue underlying the epithelial layer of the gut [7].

### 2.1. Morphological Differences between DCs and Macrophages (MΦ) in the Murine Intestine

DCs belong to the group of mononuclear phagocytes (MPs) with macrophages (MΦ) being another cell type belonging to this group. Discrimination between DCs on one hand and MΦ on the other hand is still a matter of ongoing debate. However, concerning intestinal DCs and MΦ, certain surface markers and transcription factors have been reported to be uniquely expressed by only one of these two groups. In the murine intestine, surface proteins which are exclusively expressed by DCs are CD103 [8–10], CD26, and CD272 [9]. However, CD103 is not expressed from every DC subset (see below) [11–13]. A DC specific transcription factor is Zbtb46 [13]. The only MPs in the murine intestine that express the proteins CD14, MerTK [9, 14], F4/80, and CD64 [15] are intestinal MΦ. The widely used surface markers for DC-macrophage discrimination, CD11c and MHC-II, are not useful to distinguish murine intestinal DCs from MΦ, since both proteins can be expressed in DC or macrophage subpopulations [13, 15–19]. The expression of CD11b and MHC-II varies among DC and MΦ subpopulations [13]. Therefore, the protein expression pattern of murine intestinal DCs under steady state conditions can be summarized as CD11c^+^CD103^+/−^CD11b^+/−^MHC-II^+^CD26^+^CD272^+^Zbtb46^+^CD14^−^MerTK^−^F4/80^−^CD64^−^, while the phenotype of intestinal murine MΦ is CD11c^+/−^CD103^−^CD11b^+/−^MHC-II^+/−^CD26^−^CD272^−^Zbtb46^−^CD14^+^MerTK^+^F4/80^+^CD64^+^. Another distinctive feature between DCs and MΦ is the migratory and proliferation behaviour. In general, intestinal DCs are short-lived, proliferating migratory cells while MΦ are tissue resident, long-lived, and nonproliferating [13].

### 2.2. DC Subpopulations in the Intestine

**Figure 1:**
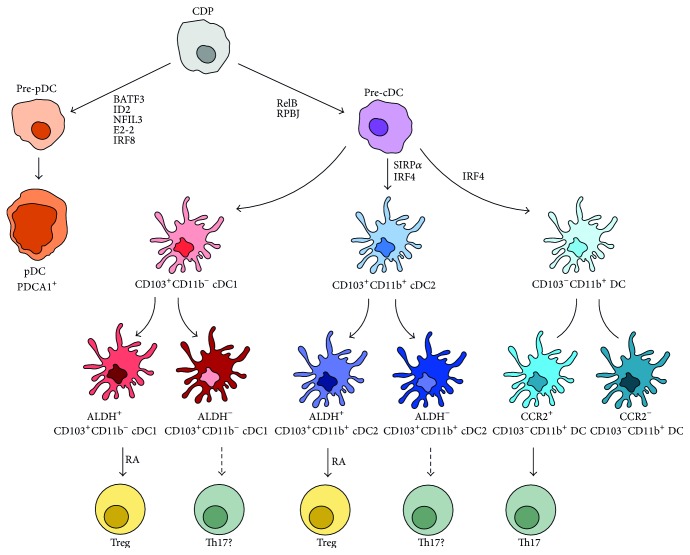
Ontogenetic development of intestinal dendritic cells subpopulations. Adapted from Guilliams et al. [35] and expanded by findings from Scott et al. [13]. See text for details. Common dendritic cell progenitor (CDP), preplasmacytoid dendritic cell (pre-pDC), precommon dendritic cell (pre-cDC), plasmacytoid dendritic cell (pDC), common dendritic cell (cDC), aldehyde dehydrogenase (ALDH), regulatory T cell (Treg), retinoic acid (RA), and T-helper 17 cell (Th17). Arrows with solid lines represent published data, and arrows with broken lines represent speculative hypotheses with missing final evidence.

As mentioned above, dendritic cells do not comprise a homogenous cell population. Different ways to distinguish one DC from another are published and popular. The most prominent approach to differentiate between distinguishable DC subsets is to focus on different expression of surface proteins, especially CD103 and CD11b [12, 13]. However, Guilliams and van de Laar have recently proposed to distinguish DCs rather by their biological function and cellular origin than their surface marker expression [11, 12]. We will adapt this system, but we will focus on DC subsets located in the intestine and add latest findings on different surface marker expression among these subsets [13]. In general, DCs derive from common dendritic cell progenitors (CDP) which, in turn, develop from hematopoietic precursor cells. CDPs may differentiate into either preplasmacytoid DCs (pre-pDCs) or precommon DCs (pre-cDCs) precursor cells [20, 21]. Murine pDCs are characterized by PDCA1 expression and their development is dependent on the transcription factors BATF3 [22], ID2 [23], NFIL3 [24], E2-2 [25], and IRF8 [22]. Murine cDCs commonly express XCR1 and SIRP*α* [26] and need RelB [27], RPBJ [28], and IRF4 [29] for differentiation. Intestinal murine cDCs additionally express CD103 and can be further subdivided into two ontogenetically different populations, dependent on their surface expression of CD11b [30]. IRF4 is needed for the CD11b^+^ lineage of these CD103-expressing or CD103-nonexpressing conventional DCs [29, 31, 32]. CD103^+^CD11b^−^ build up cDC subset 1 (cDC1) whereas CD103^+^CD11b^+^ form subgroup cDC2 (see Figure 1). One of the most important events for the maintenance of intestinal homeostasis is the induction of regulatory T cells (Tregs) (see below). Besides TGF-*β*, Treg formation is dependent on the presence of retinoic acid (RA) that is produced by dendritic cells [33, 34]. But only DCs possessing retinaldehyde dehydrogenases (ALDHs) can convert vitamin A-derived retinol to RA. Therefore, ALDH is a crucial enzyme for a subsequent induction of Tregs and thus promotion of intestinal tolerance and homeostasis. It was not clear which CD103^+^ DC subset is responsible for Treg induction, since both CD103^+^CD11b^+^ and CD103^+^CD11b^−^ DCs can produce RA and induce Tregs* in vitro* [35, 36]. Meanwhile, it could be demonstrated that each CD103^+^ DC subset (CD11b^+^ versus CD11b^−^) can be subdivided in an ALDH-expressing and a non-ALDH-expressing subset [35]. Therefore, both CD11b^+^CD103^+^ and CD11b^−^CD103^+^ DCs are able to induce a RA-mediated Treg formation. This was initially demonstrated in skin-draining lymph nodes [35], but Janelsins et al. confirmed the presence of CD103^+^CD11b^+^ALDH^+/−^ DCs also in the murine cLP [37]. Both CD103^+^ DC subsets together monitor the luminal environment in the intestine. Not only are CD103^+^ DCs able to induce Treg-mediated immune tolerance in the intestine but they are also able to promote Th17 differentiation of naïve T cells. Th17 cells contribute to the manifestation of autoimmune diseases [38, 39] and CD103^+^CD11b^+^ seem to be more efficient in Th17 promotion than their CD11b^−^ counterparts [36, 40]. It can be assumed that ALDH^−^CD103^+^CD11b^+/−^ might promote this Th17 immune response, but final evidence is missing until now.

Concerning their distribution in the intestine, CD103^+^CD11b^−^ DCs are prominent in the colonic lamina propria (cLP), while CD103^+^CD11b^+^ DCs are mostly found in the LP of the small intestine [29]. Additionally, CD103^+^CD11b^+^ DCs from the mesenteric lymph nodes (MLN) also express ALDH, which is, surprisingly, abdicable for the induction of Tregs since a loss of ALDH activity in the MLN did not affect Treg induction [29]. This might support the hypothesis that ALDH activity is more important at other intestinal sites like the lamina propria of the small or large intestine for later Treg induction after DC migration.

Recently, Scott et al. discovered an additional CD103-negative DC population in the murine intestine [13]. There is a CD11c^+^MHC-II^+^CD103^−^CD11b^+^ cell population, of which about 15% provide features of DCs like Zbtb64, CD26, and CD272 expression; they respond to Flt3L; they are migratory cells and lack macrophage markers like F4/80 and CD64 [13, 41]. They could be shown to be derived from committed pre-DCs as are CD103^+^ mucosal DCs [8]. These CD103^−^ DCs can be further subdivided into two functionally distinct subpopulations dependent on their CCR2 expression. CCR2^+^CD103^+^CD11b^+^ DCs are more efficient in Th17 induction than their CCR-negative counterparts and a loss of the CCR2^+^CD103^−^CD11b^+^ DCs leads to a defective Th17 response and therefore fails to clear a* Citrobacter* infection* in vivo*.

Another specific protein that is expressed exclusively by intestinal DCs and not by intestinal MΦ or nonintestinal DCs is SIRP*α* [42, 43]. It seems to be essential for the generation of CD103^+^CD11b^+^ since a loss of function of SIRP*α* results in a decrease of this DC population in the intestine, accompanied by markedly reduced induction of Th17 immune responses under steady-state and inflammatory conditions [42].

In general, it is important to keep in mind that CD103 expression on DCs is not a marker for universal tolerogenicity, since (1) even CD103^+^ DCs can fail to induce tolerogenicity if ALDH is not expressed and (2) a tolerogenic environment can be established even in the absence of CD103^+^ DCs [44].

### 2.3. Locations of Intestinal DCs

The murine intestine is a multifarious habitat for DCs where distinct sites harbour different DC subsets. A common feature of intestinal DCs distinguishing them from DCs from other nonintestinal tissues is the expression of CD103, with the already mentioned exception of CCR2^+/−^CD103^−^CD11b^+^ DCs, especially DCs in the small intestine (SI) and Peyer's Patches (PP) in mesenteric lymph nodes (MLN) and, with minor occurrence, in the colonic lamina propria (cLP) [8, 29, 41, 43, 45]. DCs from nonlymph node tissues remain some days at their inherent site before migrating to neighbouring draining lymph nodes [35, 46].

## 3. Antigen Sensing and Sampling by Intestinal Dendritic Cells

Invading microorganisms are recognized by pattern recognition receptors (PRRs) on the DC surface. PRRs include toll-like receptors (TLRs), retinoic acid-inducible gene I-like receptors (RLRs), and nucleotide-binding oligomerization domain-like receptors (NLRs) [47, 48]. PRRs recognize pathogen-associated molecular patterns (PAMPs) [49]. PAMPs comprise a heterogeneous class of different antigens, that is, surface components of bacteria. One of the most prominent PAMPs which usually induces DC maturation is lipopolysaccharide (LPS), an integral cell surface component of all Gram-negative bacteria. Usually, dendritic cells underlie the intestinal epithelium and therefore the connection to the colonic lumen is restricted. However, there are three prominent ways how intestinal dendritic cells can sample luminal antigens: (1) with participation of goblet cells which deliver soluble and preferably low molecular weight antigen to neighbouring DCs [50], (2) with the support of CX3CR1^+^ phagocytotic cells which can actively capture antigen followed by transport to neighbouring DCs via tight junctions [51], and (3) a direct sampling by DCs that extend their dendrites towards the lumen establishing a direct connection to the colonic lumen [52].

## 4. Intestinal Dendritic Cells and the Gut Microbiota

CD103^+^ DCs are reported to sample mainly bacteria [52] in contrast to CX3CR1^+^ MΦ which also capture soluble proteins and fungi [51, 53]. This illustrates the relevance of the bacterial microbiota composition for intestinal DCs. Interaction of DCs with the gut microbiota can occur directly by sampling bacterial antigen or by interaction with bacterial metabolic products like short chain fatty acids (SCFAs). SCFAs like butyrate can interact with the DC receptor GPR109A which finally leads to an IL-10 mediated induction of Tregs [54]. Since not all gut commensal bacteria produce SCFAs, a microbiota shift leading to dysbiosis can profoundly affect immunological mechanisms in the intestine. Toll-like receptor (TLR) signalling in DCs also seems to be crucial for the maintenance of intestinal homeostasis. Different bacterial components bind to distinct TLRs on the surface of DCs resulting in the activation of intracellular signalling cascades which leads to DC maturation or semimaturation (see below) accompanied by secretion of pro- or anti-inflammatory cytokines. The TLT adaptor molecule TNF-receptor associated factor 6 (TRAF6) seems to play a pivotal role in maintaining intestinal homeostasis since *Traf6^−*/*−^* mice fail to maintain intestinal homeostasis mediated by a reduction of Tregs and an increase of T-helper 2 (Th2) cells, finally resulting in a microbiota composition-dependent induction of colonic inflammation [55].

## 5. The Different Maturation Phenotypes of Dendritic Cells

The capability of initiating an immune response depends on the current DC maturation state. Usually, antigen encounter results in rapid DC maturation which is characterized by efficient endocytosis and antigen processing. Furthermore, upregulation of MHC-II and T cell costimulatory molecules like CD40, CD80, and CD86 enhanced expression of chemokine receptors and the secretion of proinflammatory cytokines like IL-1*β*, IL-6, TNF*α*, and IL-12 are part of DC maturation. These events influence and activate other cellular components of an induced immune response like MΦ, neutrophils, and especially T cells [56].

### 5.1. Mature DCs (mDCs)

Induction of DC maturation is accompanied by a loss of the capacity to take up and process antigen [57]. However, they literally develop into professional antigen presenting cells (APCs) indicated by powerful antigen presentation to naïve T cells [2], as well as by their ability to migrate to secondary lymphatic organs where they present antigens to T cells.

### 5.2. Immature DCs (iDCs)

Immature DCs (iDCs) express low amounts of MHC-II and T cell costimulatory molecules. They tend to promote T cell anergy and to generate Tregs, with both effects being crucial for intestinal homeostasis [58]. iDCs furthermore express high levels of PRRs with which they mediate the recognition of potential antigens and therefore their endocytosis [57].

### 5.3. Semimature DCs (smDCs) and Tolerant DCs

The definition of a semimature DC phenotype is less distinct. The most important property of smDCs uniting different definitions is the inability to induce a proinflammatory Th1 or Th17 response and to be nonresponsive, or in other words “tolerant,” towards subsequent maturation stimuli [59, 60] with the latter being the criterion that mediates the tolerogenic functions of smDCs [60]. DC semimaturation leads to a certain expression of T cell activation and a cytokine secretion pattern that is distinct from the ones of immature and mature DCs. The definite phenotype varies from one semimaturation inducing strategy to another. SmDCs that are generated by treating immature DCs with TNF*α* display a phenotype that can be summarized as CD11c^+^MHCII^hi^CD86^hi^CD80^hi^CD40^lo^CD54^+^CD205^hi^CD25^hi^TNF^lo^IL-12p40^lo^IL-10^lo^ [61]. Induction of semimaturation via low-dose LPS and subsequent dexamethasone treatment results in CD14^+^CD1a^lo^CD80^hi^CD86^hi^MHCII^hi^IL-10^hi^TNF^lo^ DCs [62]. We use a Gram-negative gut commensal,* Bacteroides vulgatus*, to induce semimaturation and define the smDC phenotype as CD11c^+^MHCII^int^CD40^lo^CD80^lo^CD86^lo^TNF*α* ^lo^IL-12^lo^IL-6^int^ [59]. Besides these strategies, DC semimaturation can be induced by treating immature DCs with ATP and LPS [63], low dose* Salmonella* LPS [64], *α*-1 antitrypsin [65],* Bacteroides fragilis* PSA [66], or* Echinococcus multilocularis* cell aggregates [67]. The resulting phenotypes concerning the most important immunomodulatory molecules are summarized in Table 1.

### 5.4. Tolerogenic DCs

While mature DCs (mDCs) promote efficient induction of inflammatory immune responses, iDCs and smDCs fail to do so. They rather have the property to actively prevent from inflammatory reactions and are therefore also termed tolerogenic DCs (tolDCs). The term “tolerogenic” includes one, several, or all of the following features DCs must provide to be considered “tolerogenic”: (1) the induction of unresponsiveness of T cells, (2) active induction of Tregs, (3) inhibition of proinflammatory T cell responses, and (4) promotion of T cell apoptosis or T cell anergy [6]. In this context, the interplay between the intestinal epithelial cells and the host immune system is of essential importance.

More generally, regulatory or tolerogenic DCs keep their ability to present antigens, but at the same time they downregulate the expression of T cell costimulatory molecules and proinflammatory cytokines but in turn upregulate inhibitory molecules like PD-L1, CD95L, or IDO as well as anti-inflammatory cytokines such as TGF-*β* and IL-10 [68]. Furthermore, they are resistant to a second maturation inducing signal [68]. Importantly, DCs also influence the differentiation of naïve T cells into Th1, Th2, Th17, or Treg cells, mostly due to supplying a certain cytokine environment [69]. In a healthy individual, the presence of tolDCs is important and a loss of tolDCs can result in the development of AID [4]. Semimature DCs are potent tolerant and tolerogenic DCs since they fulfil many to all of the above-mentioned criteria, dependent on the agent with which semimaturation is induced. As already mentioned before, the main characteristic that makes semimature DCs tolerogenic is their unresponsiveness (tolerance) towards subsequent maturating stimuli [59, 64, 65].

## 6. The Role of Dendritic Cells in Induction of Inflammatory Bowel Disease (IBD)

The development of inflammatory bowel diseases (IBD) with its two major representatives Crohn's disease (CD) and ulcerative colitis (UC) is associated with (1) an inappropriate immune response to normally benign stimuli like commensal microbes, (2) an inefficient clearance of microbes leading to a continuous stimulation of the immune system, or (3) failing to turn from an adequate proinflammatory response to inflammation resolving anti-inflammatory immune reactions [70]. In this context, the composition of the intestinal microbiota is decisive for the onset of colonic inflammation in most mouse models of experimental colitis [71] and intestinal DCs are crucial for driving immune responses in either a proinflammatory or a rather homeostatic direction [72]. For example, *Il10^−*/*−^* mice develop chronic colitis which results from the absence of suppression of MyD88-dependent commensal-induced inflammation by IL-10 [73].

Under steady-state conditions, circulating Ly6C^hi^ monocytes are repopulated into tolerogenic F4/80^low^CD103^+^CD11c^+^ LP DCs, which contribute to homeostasis by supporting tolerogenic functions [16]. On the other hand, under inflammatory conditions during colitis, Ly6C^hi^ monocytes develop into CD103^−^CX3CR1^int^CD11b^+^ LP DCs, which mediate inflammation during colitis [16].

The tolerogenic functions of intestinal DCs are mainly mediated by the induction of regulatory T cells (Tregs). As a characteristic feature, Tregs express the transcription factor forkhead box P3 (Foxp3) [74]. Induction of CD4^+^CD25^+^Foxp3^+^ Tregs is essential for intestinal homeostasis [75] and a loss of Tregs leads to a fatal multiple-organ-associated autoimmune disease [76]. Tregs are usually converted in the peripheral immune system with the help of CD103^+^ dendritic cells [77] whereupon this Treg induction is dependent on the presence of TGF-*β* and retinoic acid (RA) [78].

However, during colitis, CD103^−^CXCR1^int^CD11b^+^ DCs, although also present under steady-state conditions, massively infiltrate the colonic LP and mediate proinflammatory immune responses by producing IL-12, IL-23, iNOS, and TNF [16].

## 7. Possible Molecular Mechanisms of DC Tolerance Induction in the Intestine

**Figure 2:**
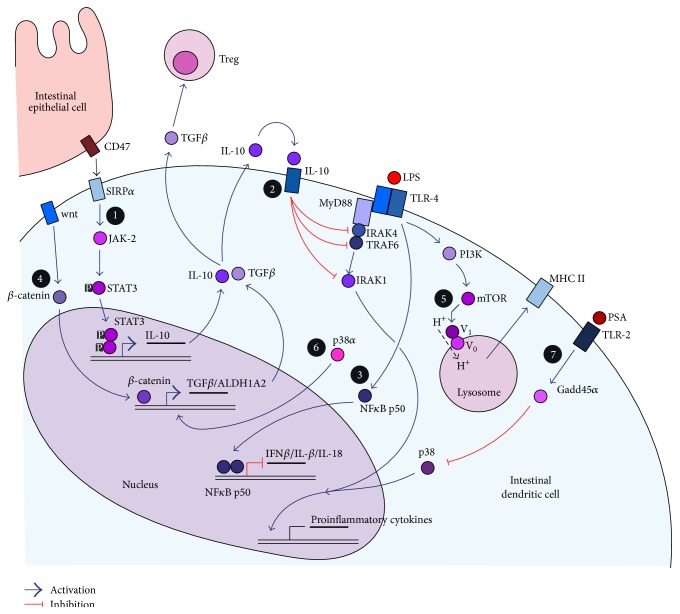
Possible molecular mechanisms of tolerance induction in intestinal dendritic cells. The white numbers in black circles refer to the numbering of regulation mechanisms in the text. See text for details.

Less is known about defined mechanisms of tolerance induction in intestinal DCs. However, knowledge about tolerance induction mechanisms of other DC subsets or of* in vitro* generated DCs can be transferred to intestinal DCs to explain how they manage to tolerate luminal bacterial or food antigens and therefore prevent from uncontrolled inflammatory reactions. Here, we want to present latest research results and discuss how and if these findings can be assigned also to intestinal DCs. All proposed mechanisms are summarized in Figure 2.

*(1) Cell-to-Cell-Contact and STAT3 Signaling*. Epithelial cells of the intestine express the surface protein CD47 which can directly interact with signal regulatory protein *α* (SIRP*α*) expressed on the surface of DCs which underlie the intestinal epithelial cell layer. This protein-protein-interaction has been shown to result in a janus kinase-2 (JAK-2) dependent signal transducer and activator of transcription 3 (STAT3) activation downstream of SIRP*α* in DCs [79]. STAT3 activation, in turn, leads to enhanced IL-10 secretion from DCs and therefore promotes tolerogenic properties in the intestinal environment [79]. STAT3 has long been known as a crucial negative regulator of immunity. Disruption of STAT3 leads to a loss of T cell tolerance in mice and efficient STAT3 signaling is associated with the immature DC phenotype, general IL-10 secretion, and tolerance induction [80]. Therefore, not only does DC alone seem to be important for homeostasis maintenance but also the “teamwork” with neighbouring epithelial cells seems to contribute to tolerance mechanisms.

*(2) IL-10 as a Central Cytokine for Intestinal Homeostasis*. Interleukin-10 is a key inhibitory cytokine in T cell activation and a mediator of intestinal homeostasis [81]. It is secreted by T cells, B cells, and most myeloid-derived cells [82]. Mice lacking functional IL-10 or its IL-10R receptor counterpart spontaneously develop severe intestinal inflammation.

Supporting the idea of IL-10 being a crucial mediator for intestinal homeostasis [83]. Also humans with defective mutations in the genes encoding for IL-10 or IL-10R develop a severe form of enterocolitis within the first months after birth [84]. These observations made IL-10 a promising therapeutic candidate in order to treat chronic inflammatory conditions of the intestine. However, results were not convincing since, in mice as well as in humans, IL-10 administration did not ameliorate the inflammatory conditions [85]. IL-10 not only affects T cell responses but can also provide autocrine and paracrine effects on DCs. Since DCs express the IL-10 receptor (IL10R), IL-10 can bind to IL10R resulting in a negative regulation of myeloid differentiation primary response 88 (MyD88) signaling inside DCs. MyD88 is an adaptor molecule of TLRs and is required for downstream TLR signalling. IL-10/IL10R interaction mediates this negative regulation by a downregulation of interleukin 1 receptor associated kinase 4 (IRAK4) on the protein level without altering IRAK4 gene transcription rates [86]. It also leads to dissociation of MyD88 from TLRs and subsequently promotes proteasomal degradation of IRAK1, IRAK4, and TRAF6, therefore silencing MyD88-dependent TLR signalling [86]. However, this is just the case if LPS as a TLR4 ligand is present at the same time to induce TLR signaling. IL-10 silencing of MyD88 signaling seems to be crucial for the maintenance of intestinal homeostasis since *IL-10^−*/*−^* mice fail to develop intestinal inflammation if these mice simultaneously lack MyD88 [73]. As a consequence, the cytokine environment does also affect DCs in their ability to induce tolerance mechanisms.

*(3) NFκB Signalling as a Mediator for Tolerogenicity*. A key regulator for DC maturation and inflammatory reactions in general is NF*κ*B [87, 88]. NF*κ*B family members do not only have an activating potential for the induction of proinflammatory cytokines. Two NF*κ*B proteins, p50 and p52, have been associated with transcription repression functions and therefore induction of tolerance [89, 90]. Both proteins lack the carboxyterminal transactivation domain and can form inhibitory homodimer complexes that prevent from transcription of proinflammatory genes. NF*κ*B p50 has been shown to promote a tolerogenic DC phenotype by negatively affecting DC survival and their capacity to efficiently activate T cells [91]. Accumulation of p50 in the nucleus of tolerogenic DCs can be accompanied by enhanced expression of tolerance-promoting molecules like indoleamine dioxygenases (IDOs) and decreased expression of proinflammatory cytokines like IFN*β*, IL-1*β*, and IL-18 [91]. These implications for p50 in the induction of tolDCs are supported by the finding that p50-deficient DCs are weak inducers of a Foxp3^+^ Treg differentiation [92]. Formation of p50-p50 homodimers contributes to LPS tolerance in MΦ [93] and p50 expression in immature DCs is crucial to prevent from autoreactive T cells [91].

*(4) β-Catenin Promotes DC Tolerogenicity*. *β*-catenin is a transcription factor and part of the wnt signalling pathway. It could be demonstrated that this signalling pathway with the subsequent release of *β*-catenin into the nucleus results in the induction of tolDCs [94]. Gene expression profiles of intestinal LP DCs revealed that this signalling pathway is decisive for the DC to become either mature or tolerogenic. *β*-catenin translocation into the DC nucleus resulted in the expression of various tolerance-associated factors like retinoic acid-metabolizing enzymes, IL-10 and TGF-*β* [94].

*(5) Prevention of V-ATPase Domain Assembly Induces Tolerogenic DCs*. Vacuolar (H^+^)-ATPases (V-ATPases) are ATP-driven proton pumps. They are composed of two domains: a peripheral V_1_ domain and membrane-embedded V_0_ domain [95]. V-ATPases are involved in acidification of lysosomes by shuffling protons from the cytosol into the lysosomic lumen [96]. The pH value of lysosomes is a crucial regulator for the efficiency of antigen processing since lysosomal proteases being involved in antigen proteolysis require acidic environments [95]. The most important mechanism to regulate lysosomal acidification is to control the assembly of the two V-ATPase domains which is a required event for forming a functional proton pump. It is known that activation of TLRs promotes domain assembly and therefore supports DC maturation [96]. Domain assembly seems to be a PI-3 kinase and mTOR mediated event since inhibitory substances for both molecules could block V-ATPase domain assembly and therefore prevent from DC maturation and promote the induction of a tolerogenic phenotype [95]. Also, stimulation of integrins and E-cadherins by cluster disruption of DC prevents from domain assembly and supports induction of a tolerogenic phenotype [96, 97].

*(6) p38α Expression Influences Expression of ALDH1A2*. MAP kinases like ERK, JNK, and p38*α* form central pathways that are activated by innate immune signals like PAMPs [98, 99] and excessive activation of MAP kinases are reported to be associated with many autoimmune and inflammatory diseases [99]. However, the MAP kinase p38*α* provides a dichotomic role. Besides being involved in promoting proinflammatory responses, its activity seems also to be crucial for the induction of a tolerogenic phenotype in intestinal CD103^+^ DCs. In these DCs, p38*α* is constitutively active and this activity is crucial for the expression of TGF-*β* and aldehyde dehydrogenase 1A2 (ALDH1A2), the latter being involved in metabolizing retinoic acid (RA). TGF-*β* and RA are involved in Treg generation and therefore promote gut homing properties of T cells [99].

*(7) Gadd45α-Mediated TLR2 Signalling Contributes to Tolerogenic Features of Intestinal DCs*. An abundant bacterial gut commensal,* B. fragilis*, is able to protect from the induction of EAE and experimental colitis and increases the proportions of CD103^+^CD11c^+^ DCs [100, 101]. It could be demonstrated that this effect is mediated by polysaccharide A (PSA), an immunomodulatory component present in outer membrane vesicles derived from* B. fragilis* bacteria [66]. PSA promotes immunological tolerance by inducing IL-10 producing Foxp3^+^ Tregs and protects animals from experimental colitis [102]. The PSA caused induction of tolDCs is dependent on TLR2 and growth arrest and DNA-damage-inducible 45 *α* (Gadd45*α*), since Gadd45*α*-deficient DCs are unable to mediate PSA-induced protection of experimental colitis [66]. Gadd45*α* itself inhibits an alternative way of MAPK p38-mediated signalling [103] and PSA-containing outer membrane vesicles lead to upregulation of Gadd45*α* [66].

Taken together, all of the mentioned molecular mechanisms of tolerance induction in DCs are potentially able to take place in the intestine, either through participation of neighbouring intestinal epithelial cells or through direct interaction of DCs with luminal content. Concerning luminal content, bacteria and their PAMPs could promote all of the potential mechanisms via interaction with host pattern PRRs, especially TLRs. We identified apathogenic Gram-negative commensal strains, namely,* Bacteroides vulgatus* mpk and* Escherichia coli* mpk, mediating completely contrary effects on DC maturation and, in consequence, the progress of experimental colitis in mice [104, 105]. As mentioned above,* B. vulgatus* interaction with immature DCs converted them into a tolerant and tolerogenic semimature phenotype characterized by low to intermediate expression of MHC-II, CD40, CD80, and CD86, almost absent secretion rates of TNF*α* and IL-12p70, and remarkable IL-6 secretion [59]. As a characteristic of tolerant DC, this phenotype could not be overcome with a subsequent maturating bacterial stimulus or by CD40 ligation [106].* E. coli* mpk stimulation, however, resulted in efficient DC maturation. As a consequence,* E. coli* mpk colonization in experimental mouse colitis using *Il2^−*/*−^* mice resulted in colonic inflammation, a feature that could be prevented by simultaneous* B. vulgatus* mpk colonization [104, 105]. We could prove that both bacteria differentially alter the phenotype of dendritic cells not only* in vitro* but also* in vivo* in the colonic LP via adjusted bacterial colonization of the gut [105]. In this context, feeding* B. vulgatus* always induced tolerant and tolerogenic DC in the colonic LP. In another study using distinct* E. coli* bacteria that just differ in the structure of their cell surface LPS, we could prove that the LPS structure alone decides if LP DCs are converted into a mature phenotype and therefore promote inflammation or if they are converted into tolerogenic semimature DCs and thus maintain intestinal homeostasis [107]. As LPS primarily signals via TLR4, tolerance induction mechanisms where NF*κ*B p50, Gadd45*α*, MyD88-signaling, *β*-catenin, and/or V-ATPase domain assembly are involved could be possible. Since LPS is a cell wall component of all Gram-negative bacteria, the resulting abundance in the intestinal lumen could largely contribute to tolerance induction in intestinal DCs. As we have demonstrated, different commensal bacteria can have opposite effects on DC maturation. This makes the composition of the microbiota decisive on whether DCs mediate tolerogenic or inflammatory LPS-triggered immune responses.

## 8. Perspectives for Clinical Approaches for the Treatment of IBD Using Tolerant and Tolerogenic Dendritic Cells

In order to be suitable as an administrable therapeutic, tolerogenic DCs (tolDCs) have to be generated* in vitro*. One efficient way to induce tolDCs is coincubating them with apoptotic cells. Phagocytosis of apoptotic cells through DCs results in production of TGF-*β* which in turn contributes to immune tolerance. Apoptotic cell-treated DCs efficiently converted naïve CD4^+^ T cells into Foxp3^+^ Tregs [108, 109]. In general, apoptotic cell induced tolDCs are important for induction of immune tolerance [110, 111]. They provide upregulation of Galectin-1 and CD205 [112], two molecules that facilitate the manifestation of immune tolerance [113, 114]. At the same time, apoptotic cell treated DCs downregulate Gr-1 and B-220 [112], two molecules triggering inflammatory responses. These DCs furthermore downregulate the transcription factor ROR*γ*t which is the decisive transcription factor for Th17 differentiation. Not only does treatment with apoptotic cells lead to induction of tolDCs but also treatment with herbal coumarins [115] and the macrocyclic antibiotic rapamycin [116] leads to tolDC induction.* In vitro* generated tolDCs have already been successfully used for the treatment of autoimmune disorders in animal models and peripheral tolerance could be restored by administrating tolerogenic DCs [117]. Approaches to treat autoimmune type 1 diabetes in a mouse model using nonobese diabetic (NOD) mice with tolerogenic DCs were very successful [112]. To do so, apoptotic islet cells were used to induce DC mediated tolerance against own islet cells [118]. All these applications lead to the question if transfer of tolerogenic dendritic cells would also be an approach to treat IBD and, if yes, which method to induce DC tolerance would be the method of choice. A published approach for the treatment of Crohn's disease patients is* in vitro* generation of DCs followed by pulsing with dexamethasone, proinflammatory cytokines IL-6, IL-1*β*, and TNF*α*, and PGE_2_ [119]. Concerning our findings that a certain gut commensal,* B. vulgatus* mpk, efficiently induces tolerant and tolerogenic DCs* in vitro* as well as* in vivo* [59, 104, 105], we would recommend using host gut commensal bacteria for* in vitro* tolDC generation. In order to provide more potential luminal antigens presented by MHC-II of tolDCs, a defined mixture of commensal bacteria could be used. This would enlarge the amount of antigenic peptides against which tolerance would be induced.

## Figures and Tables

**Table 1:** Phenotypes of semimature dendritic cells dependent on semimaturation inducing agent. LPS (lipopolysaccharide), Dex (dexamethasone), and PSA (polysaccharide A); high expression (hi), low expression (lo), intermediate expression (int), and not determined (n.d.).

Semimaturation inducing agent	MHC-II	CD40	CD80	CD86	IL-10	IL-6	TNF*α*	IL-12	Source
*B. vulgatus*	int	lo	lo	lo	n.d.	int	lo	lo	[59]
TNF*α*	hi	lo	hi	hi	lo	n.d.	lo	lo	[61]
LPS + Dex	hi	n.d.	hi	hi	hi	n.d.	lo	n.d.	[62]
*B. fragilis* PSA	int	n.d.	n.d.	n.d.	hi	n.d.	n.d.	n.d.	[66]
ATP + LPS	hi	lo	hi	hi	hi	n.d.	lo	lo	[120]
*E. multilocularis*	lo	n.d.	n.d.	lo	int	lo	n.d.	lo	[121]
Low dose LPS	int	lo	lo	lo	n.d.	int	lo	lo	[64]
*α*-1 Antitrypsin	int	lo	n.d.	int	hi	lo	n.d.	n.d.	[65]
